# Tributyltin Alters Hepatic Immune Microenvironment to ProMote Liver Fibrosis Progression in Rats

**DOI:** 10.3390/toxics14040274

**Published:** 2026-03-25

**Authors:** Nuo Zhou, Xiaoyu Si, Wenhuan Yao, Jiliang Si, Dong Cheng, Hui Li

**Affiliations:** 1 School of Public Health, Cheeloo College of Medicine, Shandong University, Jinan 250012, China; 2 College of Food and Pharmaceutical Science and Technology, Shandong Vocational Animal Science and Veterinary College, Weifang 261061, China; 3 Department of Toxicology, Shandong Center for Disease Control and Prevention, Jinan 250014, China

**Keywords:** tributyltin, liver, macrophage, niche, immunosuppression

## Abstract

Tributyltin (TBT) is well known for inducing imposex in mollusks. Studies have shown its hepatotoxicity and immunotoxicity in laboratory animals, with macrophages playing a crucial role in maintaining hepatic homeostasis and influencing disease progression; however, no research has yet explored its effects on hepatotoxicity and immunotoxicity based on hepatic macrophages. To address this gap, weaned rats were treated with corn oil or TBT (0.5, 5, or 50 μg/kg) via oral gavage every three days for 60 days. Liver sections were then subjected to hematoxylin and eosin staining, Oil Red O staining, Sirius Red staining, immunohistochemistry, and immunofluorescence to assess the effects of TBT. Hepatic function and inflammatory state were evaluated by serum biochemistry and quantitative reverse transcription-PCR (qPCR), respectively. Histological examination indicated that TBT exposure did not increase hepatic lipid accumulation but resulted in hepatocyte edema and congestion in the 5 and 50 μg/kg groups, accompanied by progressive hepatic fibrosis. In parallel, 50 μg/kg TBT increased the number of macrophages, driven by an increase in the CD206^+^CD68^+^ subset. qPCR analysis revealed a significant decrease in the expression of pro-inflammatory cytokines (such as IL-1β and TNF-α), confirming an immunosuppressive state in the livers of rats exposed to TBT. Moreover, the significant increase in serum ALT activity further revealed hepatic injury induced by 50 μg/kg TBT. In summary, TBT exposure restructures the hepatic immune microenvironment, promoting the progression of liver fibrosis independently of fat accumulation in rats.

## 1. Introduction

Tributyltin (TBT) has been extensively used as a highly effective biocide in antifouling paints for ships and shipyards [1]. Although banned in antifouling paint since 2008 due to its role in causing imposex in gastropods, TBT has continued to be detected in waters [2,3,4,5], particularly in sediments [6]. Its half-life ranges from one to five years in well-oxygenated surface sediments, but can extend to several decades in fine-grained anoxic sediments [7]. Owing to its lipophilic and ionic characteristics, TBT can bioaccumulate through the food chain, reaching elevated concentrations in marine mammals and humans [1]. One review reported an overall mean concentration of TBT in seafood of 182.33 μg/kg [8]. Human exposure primarily occurs through the consumption of aquatic products and in occupational settings [9]. One survey showed urinary tin detection frequencies of 81.05% in adults and 91.29% in children [10]. Two studies detected elevated butyltin levels in human livers obtained at autopsy [11,12]. A recent case–control study found that TBT elevated the risk (6.44-fold) of human nonsyndromic cleft lip and/or palate (NSCL/P), with median placental TBT concentrations of 8.93 μg/kg in NSCL/P cases (*n* = 109) and 5.33 μg/kg in controls (*n* = 128) [13].

TBT can cause immunotoxicity [14], hepatotoxicity [15,16,17], and hepatic steatosis in laboratory animals [18,19,20,21]. Macrophages (MΦs) play a vital role in maintaining hepatic homeostasis and influencing disease progression [22,23]. However, no research has explored the effects of TBT on hepatotoxicity and immunotoxicity based on hepatic MΦs. This study aimed to investigate the effects of TBT on the liver and identify the underlying mechanisms involving hepatic MΦs.

## 2. Materials and Methods

### 2.1. Reagents

Tributyltin chloride (CAS 1461-22-9, purity ≥ 97.0%) was obtained from TCI Shanghai Development Co., Ltd. (Shanghai, China). Serum alanine aminotransferase (ALT) and aspartate aminotransferase (AST) assay kits were purchased from Leadman Bio Co., Ltd. (Beijing, China).

Primary antibodies, including polyclonal rabbit anti-CD11c (Cat# GB11059), CD206 (Cat# GB113497), and secondary antibodies, including goat anti-mouse-HRP (Cat# GB23301), goat anti-rabbit-Alexa Fluor^®^ 488 (Cat# GB25303), goat anti-mouse-CY3 (Cat# GB21301), as well as BCA Protein Assay kits, were obtained from Wuhan Servicebio Technology Co., Ltd. (Wuhan, China). Monoclonal mouse anti-CD68 (Cat# ab31630) was obtained from Abcam Shanghai Trading Co., Ltd. (Shanghai, China). The Qiagen RNeasy^®^ Plus Mini Kit (Cat# 74134) and QuantiTect SYBR^®^ Green RT-PCR Kits were sourced from Qiagen (Qiagen, Hilden, Germany). All other chemicals used in this study were of analytical grade and were obtained from commercial sources.

### 2.2. Animals and Treatment

Weaned male Sprague-Dawley (SD) rats (about 28 days old, body weight 77.85 ± 7.36 g) were obtained from Jinan Pengyue Laboratory Animal Breeding Co., Ltd. (Jinan, China, license no. SCXK [(Lu] 20190003). Animals were housed and bred in a specific-pathogen-free environment with controlled temperature (23 °C ± 2 °C) and humidity (55% ± 15%) under a strict 12 h light cycle. All animal experiments were approved by the Ethics Committee of the School of Public Health, Shandong University. After three days of acclimatization, the rats were randomly assigned to four groups (*n* = 10 per group) according to their body weight to ensure comparable average weights across groups.

The animals received treatments of corn oil (vehicle) or TBT (0.5, 5, or 50 μg/kg) dissolved in corn oil via oral gavage every three days for 60 days, and were euthanized one day following the final gavage. The dosage and exposure frequency in this study were determined based on the FDA’s advice on fish consumption (https://www.fda.gov/food/consumers/social-media-toolkit-fdaepa-advice-about-eating-fish; accessed on 1 October 2021) and data from Ref. [8]. The FDA recommends that Americans consume fish 2 to 3 times per week, with each serving being 4 ounces. Consequently, the exposure frequency in this study was set at every three days, rather than daily.

Given an overall mean concentration of TBT in seafood of 182.33 μg/kg, the average TBT content in a 4-ounce serving of seafood is approximately 20.68 μg. Assuming an adult weighs 60 kg, the TBT intake per serving is approximately 0.345 μg/kg. Since seafood consumption is only one of the routes of TBT exposure, we selected 0.5 μg/kg as the lowest exposure dose. Subsequently, this dose was multiplied by a safety factor of 100 to obtain the maximum exposure dose of 50 μg/kg.

### 2.3. Preparation of Serum and Tissue Isolation

The rats were anesthetized with urethane (~1.2 g/kg) via intraperitoneal injection, and blood was collected from the ventral aorta as in the previous study [24]. The animals died from excessive blood loss under deep anesthesia. Each experimental group consisted of 10 animals per treatment condition unless otherwise stated. The serum was separated and stored at −80 °C for subsequent analysis. The left lobe of each animal’s liver was excised and fixed in 4% (*v*/*v*) paraformaldehyde overnight at 4 °C. The remaining liver samples were cut into smaller pieces, frozen in liquid nitrogen, and stored at −80 °C for additional analysis.

### 2.4. Histopathological Analysis

Formalin-fixed specimens were processed using standard histological techniques. After being fixed for 12 h, they were embedded in paraffin and sectioned into 4-μm-thick slices. Hematoxylin and eosin (H&E) staining and hepatobiliary pigment staining were performed using standard procedures for morphological observations. Oil Red O staining was carried out on frozen liver sections embedded in optimum cutting temperature compound according to reference [20]. Six samples were randomly selected from each group to evaluate liver fibrosis using Sirius Red staining (SRS) according to reference [25]. The tissue sections were examined under a light microscope (CX21, Olympus Corporation, Tokyo, Japan).

### 2.5. Serum Biochemistry

Serum ALT and AST levels were measured using an autoanalyzer (Beckman Coulter AU480, Tokyo, Japan) according to the manufacturer’s protocol, employing the aspartate and alanine substrate methods, respectively.

### 2.6. Immunohistochemistry (IHC)

For IHC staining, serial sections were taken from the tissue samples used for SRS (n = 6 per group). The liver sections were mounted on glass slides and incubated with primary antibodies against CD68 (1:200, at 4 °C overnight), followed by incubation with secondary antibodies for 50 min at 20 °C to 25 °C, and then stained with 3,3-diaminobenzidine. Finally, the sections were lightly counterstained with hematoxylin. Images of five different fields per sample were captured using an optical microscope (CX21, Olympus Corporation, Tokyo, Japan). Positive expressions were analyzed using the IHC Profiler program in ImageJ software (ij153-win-java8, NIH, Bethesda, MD, USA). Histochemical Score (H-Score) was used to assess the CD68 expression in each sample using the following formula: H-Score = 3 × high-positive area % + 2 × positive area % + 1 × low-positive area %.

### 2.7. Immunohistofluorescence (IHF)

Macrophage (MΦ) phenotype was further characterized using double IHF. For IHF staining, serial sections were prepared from the tissue samples that had been used for SRS (*n* = 6 per group). After dewaxing and antigen retrieval, the sections were blocked with bovine serum albumin (BSA) for 30 min at 20 °C to 25 °C. Following three washes with phosphate-buffered saline (PBS), the sections were incubated overnight at 4 °C with primary antibodies against CD68 and CD11c, or CD206 (all at a dilution of 1:200). The sections were then incubated with the corresponding fluorescently conjugated secondary antibodies (at a dilution of 1:300) at 20 °C to 25 °C for 1 h in the dark. Subsequently, the sections were counterstained with 4′,6-diamidino-2-phenylindole (DAPI). Finally, the sections were washed with PBS and mounted with an antifade mounting medium (Cat# G1401, Servicebio Technology Co., Ltd., Wuhan, China).

Images were captured from five distinct fields per sample using a fluorescence microscope (BX51, Olympus Corporation, Tokyo, Japan). Positive cells were quantified with ImageJ and normalized to DAPI-stained cells in the same field to determine the average number of positive cells.

### 2.8. RNA Extraction and Quantitative Reverse Transcription-PCR (qPCR)

Five liver samples were randomly selected from each group to isolate RNA using the Qiagen RNeasy^®^ Kit following the manufacturer’s instructions. After quality assessment, RNA was stored at −80 °C for qPCR. qPCR was conducted according to the protocol outlined in our previous study [26]. The primers used in this qPCR are listed in Table 1. Each sample was analyzed in triplicate, and target gene expression was normalized to that of β-actin in the same sample using the 2^−ΔΔ^CT method [27].

### 2.9. Statistical Analysis

All statistical analyses were conducted with IBM SPSS Statistics (version 31.0 for Windows; IBM Corp., Chicago, IL, USA). Data distribution normality was evaluated using the Shapiro–Wilk test, and Levene’s test was applied to assess the homogeneity of variances. For datasets exhibiting a normal distribution, a one-way analysis of variance (ANOVA) was performed. Following a significant ANOVA result, post hoc multiple comparisons were carried out using either the least significant difference (LSD) test or Dunnett’s T3 test, with the choice determined by the equality of variances. In cases where the data did not follow a normal distribution, the Kruskal–Wallis test was used instead. All statistical tests were two-tailed, and a *p*-value < 0.05 was considered to indicate statistical significance.

## 3. Results

### 3.1. TBT Caused No Overt Toxicity in Rats

**Figure 1:**
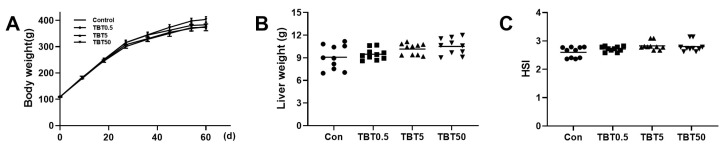
The effects of TBT on the weight of the body and liver of rats. (**A**) Body weight at all time points observed, (**B**) liver weight, or (**C**) (HSI) in rats did not alter by TBT treatment. Significance of body weight and liver weight was determined using one-way analysis of variance; Significance of hepatosomatic index was determined using the Kruskal–Wallis test. Data are presented as means ± SEM (*n* = 10 per group). HSI, hepatosomatic index; d, days.

TBT exposure did not impact the body weight of rats during the study (Figure 1A). Hepatosomatic index (HSI) is calculated as (liver weight [g]/body weight [g]) × 100. Liver weight was not significantly different between the TBT-treated groups and the control group (Figure 1B), nor was the HSI (Figure 1C).

### 3.2. TBT-Induced Hepatic Injury in Rats

**Figure 2:**
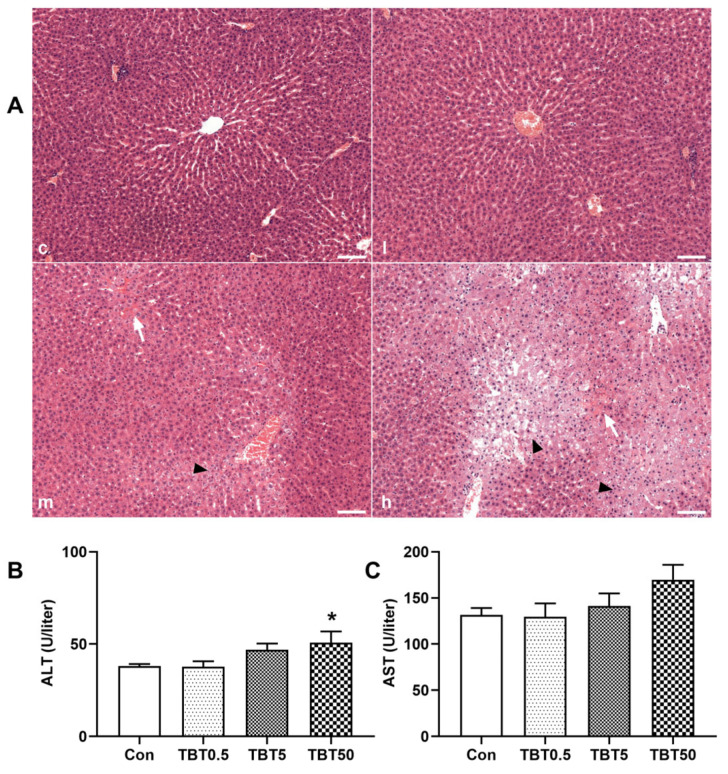
TBT treatment causing hepatic injury to rats. (**A**) Representative images of rat liver sections stained with hematoxylin and eosin; c, l, m, and h represent control, 0.5, 5, and 50 μg/kg TBT groups, respectively. The long white arrows indicate hepatic congestion, while the black arrows indicate hepatic edema; Scale bar: 100 μm; (**B**) The influence of TBT on serum ALT (**B**) and AST (**C**); Statistical significance was tested using one-way analysis of variance and then the least significant difference, * *p* < 0.05 compared to control. *n* = 10. ALT, alanine aminotransferase; AST, aspartate aminotransferase.

HE staining of liver sections is shown in Figure 2A. The extent of liver damage increased with increased doses of TBT. No significant alterations were observed in the liver sections of rats from the control and low-dose groups. Mild hepatocyte edema and congestion were noted in the medium-dose group. Furthermore, in the liver sections of the high-dose group, the lobular structure appeared distorted, the hepatic cords were disorganized, and certain areas of the liver displayed considerable hepatocyte edema and congestion.

To evaluate the impact of TBT on hepatic function, we measured serum ALT and AST activities. The serum ALT levels rose with increasing doses of TBT, showing a significant increase at the dose of 50 μg/kg (*p* = 0.023, Figure 2B). In contrast, serum AST activity was only modestly affected by TBT exposure (*p* = 0.146, Figure 2C).

### 3.3. TBT Promoted the Process of Liver Fibrosis in Rats

**Figure 3:**
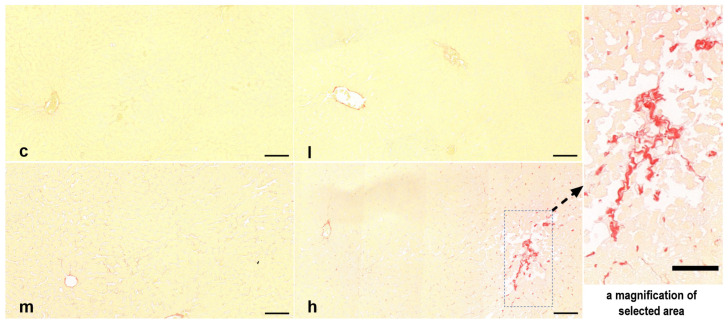
Representative images of rat liver sections stained with Picrosirius Red; Collagen fibers are visualized in red. scale bar: 50 μm for magnified area, 100 for others. c, l, m, and h represent control, 0.5, 5, and 50 μg/kg TBT groups, respectively. *n* = 6 (6 rats were randomly selected from each group).

SRS was performed to assess fibrotic collagen deposition, revealing stained areas mainly in the vasculature, with minor staining noted in the hepatic sinusoid region (Figure 3). In the control group and the low-dose group, the central vein and the portal area were stained red, but the sinusoidal spaces were relatively narrow, and the sinusoids were not clearly stained. In the middle and high-dose groups, the sinusoidal spaces were relatively enlarged, and some sinusoidal walls were stained red, forming discrete red arcs. In the high-dose group, larger fibrous red-stained areas were also found, with no obvious blood cells within the areas, indicating that this was not the central vein or the portal triad area, but rather possibly newly formed fibrous tissue.

### 3.4. Exposure to TBT Unaltered Hepatic Lipid Accumulation

**Figure 4:**
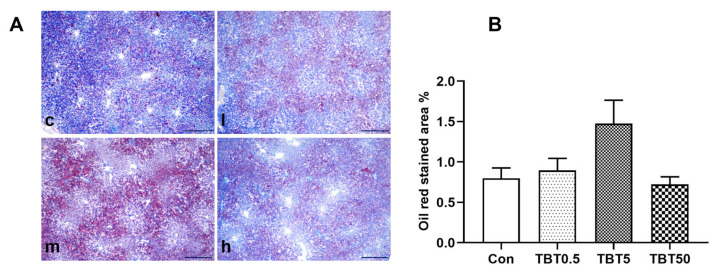
TBT unaltered hepatic lipid accumulation. (**A**) Representative Oil Red O-stained images of rat liver sections (scale bar: 500 μm). Red staining indicates the presence of lipid droplet; (**B**) the stained tissue area constituted in the hepatic sections; c, l, m, and h represent control, 0.5, 5, and 50 μg/kg TBT groups, respectively. Significance was determined using one-way analysis of variance and then the Dunnett’s T3 test, *n* = 10.

Oil Red O staining revealed that the oil droplets were primarily concentrated around the area of the triple duct and gradually tapered toward the central vein (Figure 4). There was no evidence of widespread lipid accumulation in these liver sections (Figure 4A). Furthermore, the area of stained tissue accounted for less than 2% of the corresponding liver tissue area (Figure 4B). These data suggest that TBT exposure did not cause liver fat accumulation in this study.

### 3.5. TBT Exposure Increased Hepatic MΦ Density

**Figure 5:**
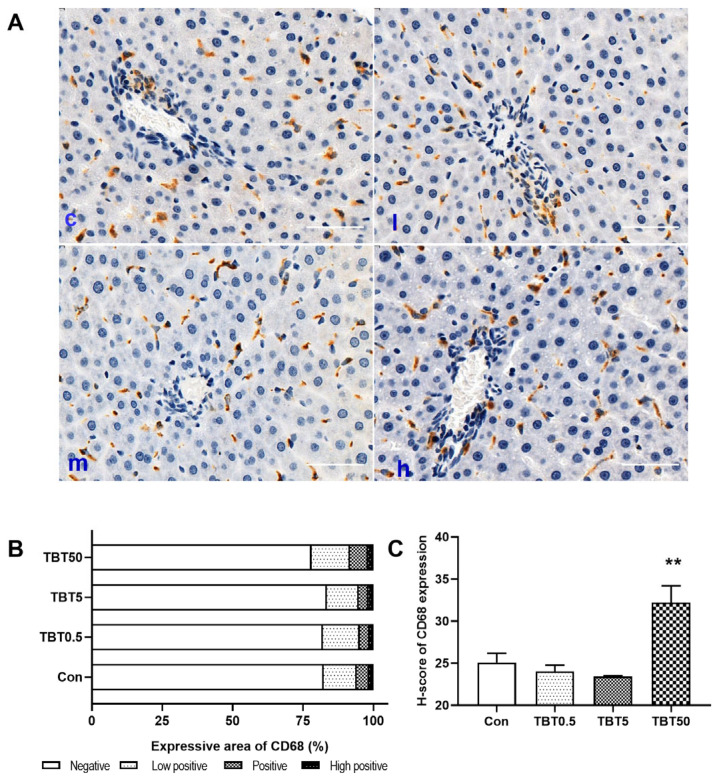
TBT increased the area of positive CD68 expression. (**A**) Representative IHC images of rat liver sections for CD68 (brown); scale bar: 50 μm. (**B**) The area and (**C**) H-score of CD68 expression in rat liver sections. Statistical significance was tested using one-way analysis of variance, and then the least significant difference, ** *p* < 0.01, compared with the control. c, l, m, and h represent control, 0.5, 5, and 50 μg/kg TBT groups, respectively. *n* = 6 (The slices were from the same rats as Figure 4). H-Score, histochemical score.

Given that MΦs play essential roles in the disease onset or progression [22], we evaluated the MΦ population in the liver using the specific marker CD68 [28]. The majority of CD68^+^ cells were stellate or spindle-shaped and preferentially localized along the sinusoidal area of the periportal zone (Figure 5A). Figure 5B depicts the expression of CD68 in different treatment groups. Quantitative analysis revealed that the H-score significantly increased by 28.48% in the 50 μg/kg TBT-treated group compared to the control group (*p* = 0.003; Figure 5C), indicating that TBT increases the number of hepatic MΦs in rats.

### 3.6. TBT Unalters M1Φs, but Increases M2Φs in Hepatic Tissue of Rats

**Figure 6:**
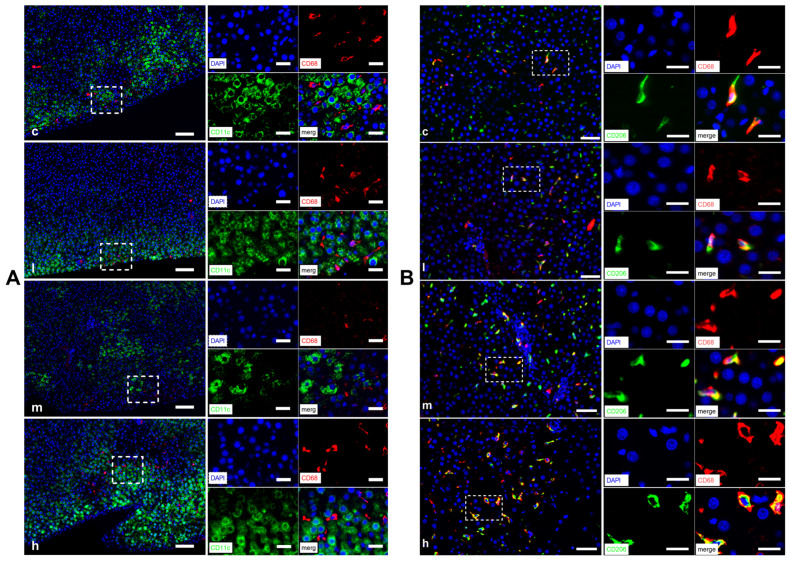
TBT increases the expression of CD206^+^CD68^+^ cells in the liver of rats. (**A**) Representative IHF image showing the distribution of M1Φ within the liver (left) and amplifying selected region (right; immunofluorescence staining for DAPI [blue], CD68 [red], CD11c [green] and their merged image). (**B**) Representative IHF image showing the distribution of M2Φ within the liver (left) and amplifying selected region (right; immunofluorescence staining for DAPI [blue], CD68 [red], CD206 [green] and their merged image). scale bars: 50 μm (left) and 20 μm (right); c, l, m and h represent control, 0.5, 5 and 50 μg/kg TBT groups, respectively. M1Φ, M1-type macrophage; M2Φ, M2-type macrophage; and DAPI, 4′,6-diamidino-2-phenylindole.

MΦs can be categorized into two primary populations: the classically activated M1-type MΦs (M1Φs) and the alternatively activated M2-type MΦs (M2Φs) [29]. Figure 6 shows the expression of macrophage markers. Using double IHF staining, we analyzed the hepatic sections for the expression of CD11c and CD206, which are surface markers for M1Φs and M2Φs [30], respectively, using double IHF staining. Notably, there was almost no overlap between cells stained with CD11c^+^ and those stained with CD68^+^ (Figure 6A), indicating that these cells are likely not M1Φs. These findings demonstrate that TBT has no impact on the proportion of hepatic M1Φs in rats.

**Figure 7:**
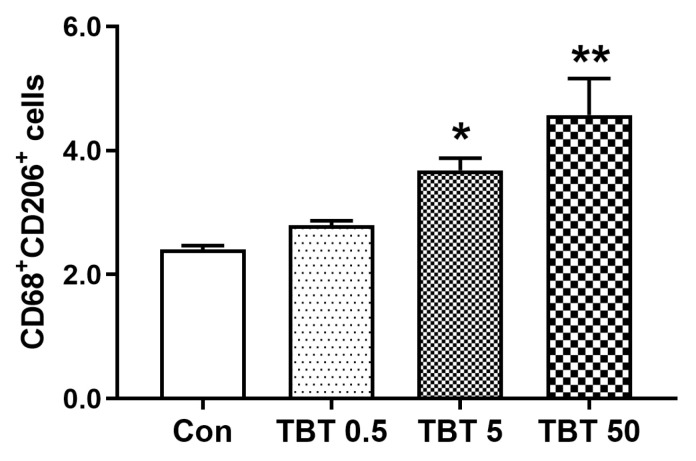
TBT elevated the number (%) of CD206^+^CD68^+^ cells in the rat liver sections. Statistical significance was tested using one-way analysis of variance and then the least significant difference, * *p* < 0.05, ** *p* < 0.01, compared with control, *n* = 6 (The slices were from the same rats as Figure 4).

CD206^+^ cells were enriched in the periportal regions and along the liver lobules. To quantify M2Φs, we selected comparable areas in all liver sections to assess the co-localization of CD206 and CD68 (Figure 6B). Quantitative analysis revealed that the number of CD206^+^CD68^+^ cells increased in dose-dependent rats exposed to TBT (*p* = 0.005; Figure 7), suggesting that TBT increases the proportion of M2Φs in rats.

### 3.7. TBT Down-Regulates the Gene Expression of Inflammation-Related Cytokines and Chemokines in the Livers of Rats

**Figure 8:**
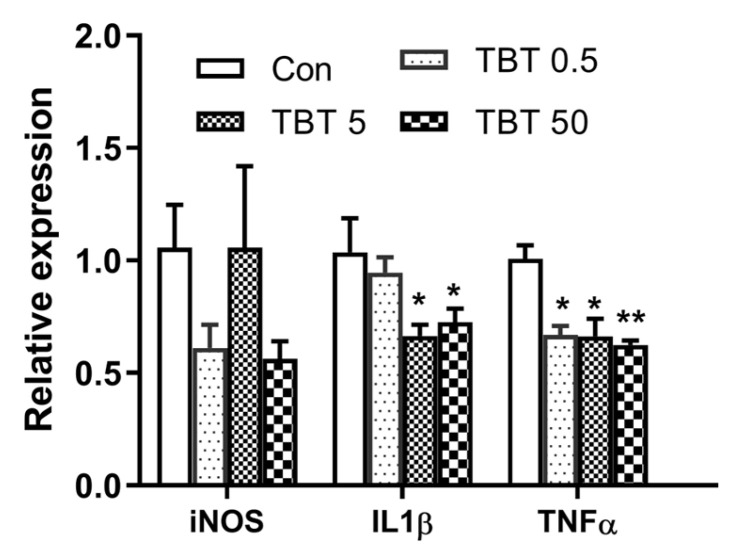
TBT down-regulated the gene expression of i representative markers of inflammation in the livers of rats. Statistical significance was tested using one-way analysis of variance, and then the least significant difference for IL-1β, Dunnett’s T3 test for iNOS and TNF-α, * *p* < 0.05, ** *p* < 0.01 compared with control. *n* = 5 (five rats were randomly selected from each group). iNOS, inducible nitric-oxide synthase; IL-1β, interleukin-1β; and TNF-α, tumor necrosis factor-α.

Inducible nitric-oxide synthase (iNOS), interleukin-1β (IL-1β), and tumor necrosis factor-α (TNF-α) are representative markers of inflammation [31]. We assessed the effects of TBT on liver inflammation by measuring the gene expression of these markers using qPCR. TBT treatment significantly decreased the gene expression of IL-1β (*p* < 0.05) and TNF-α (*p* < 0.001), but did not affect iNOS (Figure 8).

## 4. Discussion

Compared with the control group, treatment with 50 μg/kg TBT induced hepatic injury in rats, characterized by hepatocyte edema, congestion and elevated serum ALT levels. This injury may be attributed to the activation of the TBT -induced apoptotic [17,32] and autophagy pathways [33]. Consistent with our findings, a previous study reported that a single injection of one-quarter the LD50 of TBT increased serum ALT and AST levels in SD rats, accompanied by expanded hepatic sinusoids and swollen mitochondria in hepatocytes [15]. The observed mitochondrial swelling may provide a plausible explanation for the hepatocyte edema seen in our study.

Interestingly, despite these signs of injury, TBT exposure did not cause significant hepatic fat accumulation in rats. This contrasts with numerous studies reporting that low-dose TBT promotes fat accumulation in the livers of mice [19,20,21]. For instance, studies demonstrated that ancestral exposure to TBT led to hepatic lipid accumulation in unexposed offspring mice [19] via epigenetic modification [34,35]. These disparate findings suggest that species differences and variations in exposure paradigms—including the timing, route, and frequency of exposure—may critically influence the manifestation of hepatic steatosis. Indeed, acute oral toxicity experiments have shown that TBT induces more severe liver damage in mice than in rats and guinea pigs, underscoring the species-specific nature of TBT hepatotoxicity [16].

A recent study indicated that prenatal exposure to 50 nM TBT predisposed male offspring to more severe hepatic fibrosis and inflammatory responses upon subsequent challenge with a Western diet [35]. In the present study, with increasing doses of TBT, we observed a progressive increase in hepatic injury and liver fibrosis rather than in hepatic lipid levels compared to their control counterparts [35].

Liver fibrosis is a wound-healing process closely linked to tissue regeneration, orchestrated by various cell types, including macrophages [22,36]. The phenotype and function of these macrophages are critically shaped by their microenvironment [23]. In the present study, TBT exposure significantly reduced the expression of inflammatory genes in the liver, indicating that the hepatic immune microenvironment had shifted to a less inflammatory state. This TBT-induced restructuring of the immune niche may underlie the observed phenotypic changes in hepatic macrophages and contribute to the progression of liver fibrosis.

Correspondingly, we observed almost no M1Φs in the livers of rats from either the control or TBT-treated groups. At steady state, monocytes do not express CD11c, and CD11c expression was minimal in hepatic MΦs [37]. This indicates that TBT treatment did not trigger an inflammatory response. In line with our findings, a previous in vitro study demonstrated that TBT exposure did not elicit an inflammatory response in either LPS-primed or non-LPS-primed cells after a 6 h exposure to 30 μM TBT [38]. Furthermore, transcriptomic analyses in two extensive studies on TBT revealed no overexpression of inflammatory genes in either visceral fat or the liver [34,39].

In contrast to the M1 population, we observed a dose-dependent increase in M2Φs in the livers of TBT-treated rats. Given that M2Φs are involved in tissue repair and remodeling [40], this increase may represent a feedback mechanism aimed at autogenous tissue repair following TBT-induced injury. Importantly, this expansion of M2Φs did not result from the transformation of M1Φs, suggesting that the activation states of hepatic MΦs are more complex than the traditional M1/M2 dichotomy model [41]. It is widely accepted that the onset and progression of metabolic-associated fatty liver disease (MAFLD) are associated with hepatic steatosis and chronic, low-grade inflammation [42], in which M1Φs play a crucial role [43]. However, in the present study, we observed an immunosuppressive state rather than inflammation in the livers of TBT-treated rats.

A previous study indicated that type 2 cytokines facilitated the progression of MAFLD [25]. In the current study, we observed an immunosuppressive status rather than inflammation in the livers of TBT- treated rats, suggesting a distinct pathological mechanism. This immunosuppressive state may be mechanistically linked to TBT’s role as an ATP synthase inhibitor. TBT can reduce cellular ATP levels [44,45], and given that ATP is required for both the maturation and release of IL-1β and for the activation of the NALP3 inflammasome [46], the observed decrease in IL-1β expression may be a direct consequence of TBT-induced ATP depletion in the liver.

Immunosuppression has significant pathological implications, potentially increasing susceptibility to infections and even tumor development. Supporting this concern, one study demonstrated that perinatal exposure to 0.5 mg/kg TBT increased the proportion of offspring developing hepatic adenomas (at 45 weeks post-birth) [39]. Furthermore, given that over 90% of hepatocellular carcinoma cases arise in the context of fibrosis or cirrhosis [47], the progressive hepatic fibrosis observed in our study warrants particular attention. Notably, TBT-induced hepatic fibrosis occurred in the absence of steatosis, diverging from the classical paradigm of MAFLD. This unique pathological feature may offer valuable new insights into the mechanisms of TBT-induced hepatotoxicity and highlights the need for increased awareness of its immunosuppressive and pro-fibrotic effects.

This study has several limitations. This study has several limitations. First, only male rats were included, which precludes assessment of potential sex differences in TBT-induced hepatotoxicity or liver fibrosis. This is a notable gap, as recent evidence indicates that TBT exposure leads to sex-dimorphic hepatic effects [35,48], with male mice exhibiting greater susceptibility associated with more pronounced chromatin accessibility and transcriptomic changes compared to females [48].

Second, the analysis was restricted to a single time point. This limitation is highlighted by a discrepancy between our current findings and previous work. Although we observed a dose-dependent increase in lipid accumulation in the bone marrow of the femur under identical treatment conditions [49], no hepatic fat accumulation was detected in this study. This tissue-specific difference may reflect divergent temporal dynamics in response to TBT, which the current experimental design could not capture. Consequently, the use of a single sex and a solitary time point limits both the generalizability of our findings and our understanding of the temporal progression of TBT-induced liver toxicity.

## 5. Conclusions

TBT exposure in rats results in hepatic injury characterized by hepatocyte edema, congestion, and progressive fibrosis, notably without promoting hepatic lipid accumulation. This fi-brotic response is accompanied by an increase in the total number of hepatic macrophages—particularly the CD206^+^CD68^+^ M2-like subset—and a downregulation of pro-inflammatory cytokine gene expression, indicating the establishment of an immu-nosuppressive hepatic microenvironment. Thus, TBT-induced liver fibrosis, occurring independently of steatosis, appears to be driven by immune modulation via macrophages, thereby challenging prior assumptions about TBT hepatotoxicity. These findings provide valuable insights into the mechanisms underlying TBT-induced liver injury.

## Figures and Tables

**Table 1:** Primers used for qPCR analysis of gene expression.

Gene	Primer Seq (5′-3′)	Amplicon Size (bp)	GenBank
iNOS	F AGCCCTGGAAGACCCACATCTG	122	S71597.1
	R AGCCATGACCTTCCGCATTAGC		
TNF-α	F GGACACCATGAGCACGGAAAGC	133	NM_012675.3
	R CGCCACGAGCAGGAATGAGAAG		
IL-1β	F TGTTTCCCTCCCTGCCTCTGAC	110	NM_031512.2
	R CGACAATGCTGCCTCGTGACC		
β-actin	F CACTATCGGCAATGAGCGGTTCC	154	NM_031144.3
	R CAGCACTGTGTTGGCATAGAGGTC		

Note: F, forward; R, reverse.

## Data Availability

The data presented in this study are available on request from the corresponding authors.

## Abbreviations

The following abbreviations are used in this manuscript:

TBT	Tributyltin
qPCR	Quantitative reverse transcription-PCR
NSCL/P	nonsyndromic cleft lip and/or palate
ALT	alanine aminotransferase
AST	aspartate aminotransferase
HE	Hematoxylin and eosin
SRS	Sirius Red staining
IHC	Immunohistochemistry
H-Score	Histochemical Score
PBS	phosphate-buffered saline
MΦ	Macrophage
DAPI	4′,6-diamidino-2-phenylindole
F	forward
R	reverse
ANOVA	one-way analysis of variance
HSI	hepatosomatic index
iNOS	inducible nitric-oxide synthase
IL-1β	interleukin-1β
TNF-α	tumor necrosis factor-α
MAFLD	Metabolic-associated fatty liver disease
